# Macroeconomic Development and Dramatic Increase in Stroke Burden in Rural China: A 25-Year Population-Based Study

**DOI:** 10.3389/fneur.2020.00385

**Published:** 2020-05-13

**Authors:** Peng Zhao, Jie Liu, Yuhan Hao, Qiuxing Lin, Ying Gao, Jun Tu, Jinghua Wang, Yaogang Wang, Xianjia Ning

**Affiliations:** ^1^Department of Neurology, Tianjin Medical University General Hospital, Tianjin, China; ^2^Laboratory of Epidemiology, Tianjin Neurological Institute, Tianjin, China; ^3^Key Laboratory of Post-Neuroinjury Neuro-repair and Regeneration in Central Nervous System, Ministry of Education and Tianjin City, Tianjin Neurological Institute, Tianjin, China; ^4^Health Management Centre, Tianjin Medical University General Hospital, Tianjin, China; ^5^School of Public Health, Tianjin Medical University, Tianjin, China

**Keywords:** stroke, macroeconomic development, epidemiology, incidence, trends

## Abstract

Low socioeconomic status is associated with a high stroke risk. However, few studies have quantitatively assessed the relationship between stroke burden and national economic development indicators. We explored the quantitative association between macroeconomic development and stroke burden in rural China. In this population-based, prospective study (1992–2016), we collected data on annual registrations of stroke events and deaths in Tianjin, China. Economic development over the period was represented by gross domestic product annually adjusted for purchasing power parity (PPP-aGDP) and per capita net income (PCNI) of rural residents in China. We assessed the association of first-ever stroke incidence with PPP-aGDP and PCNI. During the 25-year study period, there were 1,185 stroke events and 362,296 person years of surveillance. First-ever stroke incidence increased by an average of 10.7% per 1,000 USD increase in overall PPP-aGDP and by 12.0% per 1,000 Yuan increase in PCNI; respectively, the mean increases were 9.6 and 10.8% in men and 13.0 and 14.4% in women (all, *P* < 0.001). These same changes in PPP-aGDP and PCNI also resulted in increases in the incidence of ischemic stroke (12.6 and 14.3%, respectively; *P* < 0.05), and intracerebral hemorrhage (both, 6.2%; *P* < 0.05). Similarly, in men, the age of onset of intracerebral hemorrhage decreased by 0.96-years (*P* = 0.002) for each 1,000 USD increase in PPP-aGDP and by 1.08-years (*P* = 0.003) for each 1,000 Yuan increase in PCNI. Macroeconomic development was positively associated with stroke incidence in rural China. Thus, enhancing health-care investments is crucial for containing the stroke burden during this remarkable economic development in China. Our findings could guide other developing countries with information regarding the timely control of stroke risk factors and reductions in stroke burden during the initial stages of economic development.

## Introduction

The global stroke burden continues to increase in both developed and developing countries (1, 2). Over a 23-year study period (1990–2013), there were an estimated 4.85 million stroke deaths and a loss of 91.4 million disability-adjusted life years (DALYs) in developing countries, and a corresponding 1.6 million deaths and a loss of 21.5 million DALYs in high-income countries. The developing countries have significantly greater increases in stroke burden than do developed countries (3).

Over the past three decades, the world economy has experienced rapid growth. During this period, China has experienced unprecedented development, with a gross domestic product (GDP) increase of 8.5% between 1978 and 2016 (4). Simultaneously, China has been transitioning from an agricultural society into an industrial society. As the world's largest developing country, China has also experienced strokes become the leading cause of death (5, 6), accounting for almost one-third of global stroke deaths (7). Furthermore, in contrast to the downward trend in stroke incidence in developed countries, stroke incidence has dramatically increased in China (8, 9), especially in rural areas (10). In a previous study, we reported the increased incidence of first-ever stroke among rural residents in China. Specifically, we reported an overall 6.5% increase in first-ever stroke incidence between 1992 and 2012 (11), including an 11.9% increase among adults aged 35–64-years (12). Moreover, the mean age of onset of intracerebral hemorrhage (ICH) in men decreased by 0.56-years annually (13).

Previous studies have established that stroke risk is associated with socioeconomic status (14–17) and national macroeconomic variables (18). However, few studies have assessed the magnitude of this association within a society. Thus, we investigated the quantitative association of macroeconomic development, measured as per capita GDP and per capita net income (PCNI) in Chinese Yuan, with the trend in stroke burden, represented by stroke incidence and age at onset over time in a rural population in China.

## Methods

### Study Population

This was a prospective study; its design and study population were previously described (10–12). Briefly, we selected the population of Yangjinzhuang, a township in Tianjin, China, to survey epidemiological trends in first-ever stroke incidence. Yangjinzhuang is a township of Jizhou district in Tianjin, China, with 15,438 residents, where 95% of the adults were low-income farmers. The primary source of income was grain production, and the annual per capita income was <100 USD in 1990 and <2,000 USD in 2015 (19). In 1991, the illiteracy rates among residents aged 35–74-years were 30% (men) and 40% (women). The population characteristics remained stable over the study period (20). For this study, we included all registered individuals living in Yangjinzhuang and collected data on stroke events and all-cause deaths registered between 1992 and 2016. Distribution of population by age and sex in the Tianjin Brain Study during 1992 to 2016 was showed in Supplemental Table 1.

The study protocol was approved by the ethics committee of Tianjin Medical University General Hospital (TMUGH), and written informed consent was obtained from each participant.

### Information and Processes

During the surveillance period, all stroke events and all-cause deaths were registered and followed. In addition, all changes in demographic information were recorded for each calendar year. All stroke events occurring during the study period were registered.

Stroke events were reported according to predefined procedures. The method used for ascertaining stroke events was previously described (21). Briefly, information regarding stroke events was obtained from three sources: the local, licensed, village doctors who reported according to a predetermined procedure; medical records for hospital inpatients; and the all-cause death registry. The village doctors reported stroke events to physicians in the community hospital within 24 h of onset. For outpatients, physicians visited the patients at home, to confirm stroke events within 72 h of stroke onset. Finally, all reported stroke events were biannually verified by senior neurologists using a door-to-door survey.

The diagnosis of stroke events among patients with no imaging data was confirmed via clinical examinations of the non-hospitalized patient, and medical records of hospitalized patients were reviewed by a senior neurologist of TMUGH after identification by the Quality Control Group (consisting of senior epidemiologists and neurologists from Department of Neurology in TMUGH). The medical records were obtained from the hospital or insurance company for inpatients and from family members of patients for outpatients. The third, the all-cause death registry supplemented data regarding stroke events that were not otherwise reported.

To ensure accurate recording of stroke events, members of the Quality Control Group, trained village doctors, and community hospital physicians involved with the study protocol conducted an annual survey of multiple overlapping sources, including the hospital admissions registry, local death registry, and interviews with patients' relatives. This was conducted to confirm the details for any patient whose stroke events may not have been reported. Moreover, to ensure accurate recording of stroke events, the village doctors and community hospital physicians underwent annual study protocol training by a neurologist.

### Stroke Event Definition

First-ever strokes were defined as the first occurrences (no history of stroke in prior medical records) of rapidly developing signs of focal neurologic disturbances of presumed vascular etiology lasting >24 h (22). All stroke events (intracerebral hemorrhage [ICH] and ischemic stroke [IS]) in this study were symptomatic and were diagnosed using pre-established clinical features and imaging criteria. Patients were diagnosed as having full clinical strokes if they demonstrated significant clinical symptoms and signs. Subarachnoid hemorrhage (SAH), transient ischemic attacks (TIA), and silent strokes (diagnosed by imaging only) were excluded, but stroke patients with prior histories of TIAs were regarded as incident events. Patients with transient symptoms and concurrent neuroimaging evidence of a brain infarction were also considered as stroke cases, based on the “tissue” definition (23).

### GDP (in USD) and PCNI

We used per capita GDP adjusted for purchasing power parity (PPP-aGDP), in China, expressed in constant 2011 USD, as the macroeconomic development indicator (4). The PPP-aGDP, expressed in USD, is the most commonly used indicator of economic development. This indicator avoids potential bias due to monetary exchange rates between countries and allows comparisons between studies. PPP-aGDP is expressed in constant 2011 USD to neutralize inflation effects when comparing studies from different years. Additionally, we used the PCNI (in Chinese Yuan [RMB]) of rural residents in China to represent the purchasing capacity of the individual income (24). The conversion rate of exchanged USD for RMB is available in the Supplemental Table 2.

### Statistical Analysis

First-ever stroke incidence, per person year, was calculated using the annual numbers of reported stroke patients. Age-standardized incidence rates of first-ever stroke were calculated using the direct method and the world standard population (25). Age-specific stroke incidence during the study period was estimated according to three age groups: <45-, 45–64-, and ≥65-years. Age-standardized trends in stroke incidence were assessed as annual percentage changes using the following regression model: Ln (r_t_) = a + bt, where Ln denotes the natural logarithm and t is the year. The trend, b, was estimated using ordinary regression; 100 × b represented the estimated annual percentage change in incidence. The associations of PPP-aGDP and PCNI with stroke incidence and age at stroke onset were analyzed using a linear regression model. Changes in first-ever stroke incidence were presented as partial regression coefficients (β) with standard errors; β was defined as the percentage change in stroke incidence and change in age of stroke onset per 1,000 USD increase in PPP-aGDP or 1,000 Yuan increase in PCNI. Statistical significance was defined as *P* < 0.05. SPSS, version 19.0 for Windows (SPSS, Chicago, IL, USA), was used for all analyses. In this study, we analyzed first-ever stroke incidence beginning in 1992, when new diagnostic imaging techniques were available for the study population.

## Results

### Characteristics of First-Ever Stroke Patients

**Figure 1:**
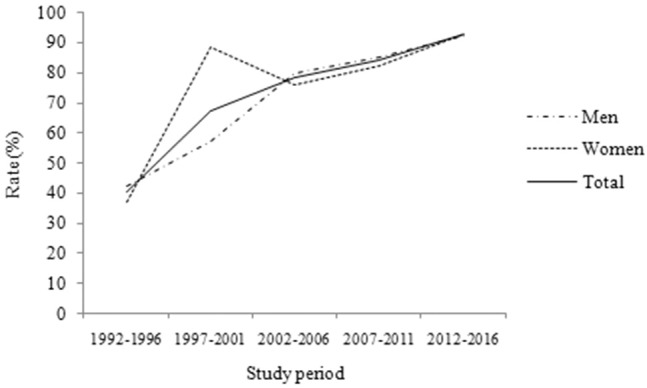
The proportion of diagnosis using neuroimaging over time during the 25-year study period. It shows that the rate of diagnosis using neuroimaging increased both in men and in women during the 25-year study period (*P* < 0.001).

Between 1992 and 2016, there were 1,185 stroke events (60% in men) and a total of 362,296 person years of surveillance. ICH was diagnosed in 22.4% of patients; IS was diagnosed in 77.6%. The average age at stroke onset was 65-years, and the age-based proportions were <45-years, 4.5% of cases; 45–64-years, 43.4%; and ≥65-years, 52.1%. Overall, 79.7% of cases were diagnosed using imaging results, including 82.3% of ICH cases and 79% of IS cases (Table 1). Moreover, the rate of neuroimaging-based diagnoses increased for both sexes over the study period (*P* < 0.001; Figure 1).

**Table 1:** Demographical characteristics among patients with first-ever stroke in the Tianjin Brain Study during 1992 to 2016.

**Characteristics**	**Total**	**Men**	**Women**
Person-year	362,296	188,622	173,674
**Case**, ***n*** **(%)**			
ICH^†^	266 (22.4)	159 (22.5)	107 (22.4)
IS^†^	919 (77.6)	548 (77.5)	371 (77.6)
Total	1185 (100)	707 (100)	478 (100)
Age, years, mean (SD)	65.35 (11.61)	65.24 (11.28)	65.52 (12.09)
**Age group**, ***n*** **(%)**			
<45-years	53 (4.5)	29 (4.1)	24 (5.0)
45 to 64-years	514 (43.4)	309 (43.7)	205 (42.9)
≥65-years	618 (52.1)	369 (52.2)	249 (52.1)
**Diagnosis by imaging**, ***n*** **(%)**			
ICH^†^	219 (82.3)	129 (81.1)	90 (84.1)
IS^†^	726 (79.0)	431 (78.6)	295 (79.5)
Total	945 (79.7)	560 (79.2)	385 (80.5)

† *ICH, intracerebral hemorrhage; IS, ischemic stroke*.

### Age-Standardized Incidence of First-Ever Stroke From 1992 to 2016 by Sex and Type

Table 2 shows the age-standardized incidence of first-ever stroke per 100,000 person years. The overall age-standardized incidence of first-ever stroke per 100,000 person years increased from 98.0 in 1992 to 451.4 in 2016. In men, the incidence was 118 in 1992 and 543.6 in 2016; in women, it was 77.9 in 1992 and 376.4 in 2016. Moreover, the age-standardized incidence of IS per 100,000 person years increased from 56.4 in 1992 to 395.9 in 2016 over the 25-year study period: 62.6 to 462.8 in men and 50.2 to 332.6 in women. Simultaneously, the overall incidence of ICH increased from 41.7 in 1992 to 55.5 in 2016: 55.4 to 80.8 in men and 27.7 to 43.8 in women.

**Table 2:** The age-standardized incidence of stroke in study population during 1992 to 2016 by sex and type.

**Year**	**Total**			**IS**			**ICH**		
	**Men**	**Women**	**Total**	**Men**	**Women**	**Total**	**Men**	**Women**	**Total**
1992	118.0	77.9	98.0	62.6	50.2	56.4	55.4	27.7	41.7
1993	179.6	66.8	122.6	102.8	35.7	68.6	76.4	31.1	48.8
1994	114.0	80.5	96.4	83.9	80.5	82.0	30.0	0	14.3
1995	185.5	101.0	157.0	131.6	104.4	118.4	41.9	26.6	33.2
1996	229.2	123.5	185.8	125.5	82.7	103.0	91.9	40.9	65.9
1997	165.0	48.6	105.0	119.6	39.5	77.8	46.4	9.1	27.3
1998	168.7	83.2	121.4	124.5	42.5	83.1	44.2	26.9	34.7
1999	178.2	62.0	118.1	114.4	38.1	74.4	63.7	23.9	36.9
2000	215.2	174.9	193.5	197.1	133.5	164.0	18.1	30.6	24.3
2001	189.2	88.0	136.3	149.7	68.2	107.2	11.2	9.1	10.1
2002	184.4	177.2	179.1	136.1	101.4	117.9	37.3	75.8	56.2
2003	329.9	168.5	257.0	267.1	97.2	181.6	78.2	71.3	74.1
2004	250.6	196.4	220.1	174.7	132.6	150.6	67.8	63.8	65.8
2005	216.8	156.7	181.4	173.1	135.9	149.7	42.9	20.2	32.3
2006	207.2	94.0	148.5	147.6	79.8	113.2	48.9	14.2	30.5
2007	364.9	342.0	353.2	254.0	243.1	230.0	100.5	90.2	93.5
2008	464.7	224.3	338.8	375.7	183.1	275.1	87.4	32.3	58.9
2009	437.6	363.3	398.1	309.9	293.7	301.1	127.7	69.6	97.0
2010	442.0	261.9	352.6	340.9	153.2	245.9	101.2	99.9	101.9
2011	383.3	251.6	315.7	282.9	211.5	246.7	100.4	40.4	69.2
2012	327.1	314.3	320.4	247.1	223.7	235.8	80.0	90.6	84.7
2013	613.0	344.8	475.4	537.4	280.8	408.7	75.6	64.1	66.7
2014	280.0	308.8	297.4	208.2	266.0	238.8	71.9	42.8	58.6
2015	373.1	217.8	291.5	332.9	175.9	250.4	40.2	41.9	41.0
2016	543.6	376.4	451.4	462.8	332.6	395.9	80.7	43.8	55.5

### Trends in Age-Standardized Incidence of First-Ever Stroke by Sex, Age, and Type

The overall age-standardized incidence of first-ever stroke per 100,000 person years increased by 6.0% annually; the increase was 7.3% in men and 5.4% in women (all *P* < 0.001). Moreover, the age-standardized incidence of IS increased significantly by 7.1% annually over the 25-year study period. Simultaneously, the incidence of ICH increased significantly by 3.6% annually. Concurrently, stroke incidence increased significantly across the three defined age groups. The increases in IS incidence were most notable in patients aged 45–64- and ≥65-years; for ICH incidence, the increases were most notable in patients aged <45- and 45–64-years. However, there was no significant change in IS incidence rate among patients aged <45-years; similarly, the ICH incidence rate did not demonstrate a significant increase among patients aged ≥65-years (Table 3).

**Table 3:** Annual proportion of changes in the age-standardized incidence of first-ever stroke by subtype and gender from 1992 to 2016.

**Groups**	**ICH**	**IS**	**Total**
**Overall**			
Men	2.6 (0.1, 5.0)^*^	6.6 (5.0, 8.1)^*^	5.4 (4.0, 6.8)^*^
Women	4.0 (0.7, 7.4)^*^	8.0 (5.8, 10.1)^*^	7.3 (5.3, 9.2)^*^
Total	3.6 (1.2, 6.0)^*^	7.1 (5.7, 8.5)^*^	6.0 (4.6, 7.4)^*^
**<45 yrs**			
Men	3.1 (−1.7, 7.9)	0.9 (−6.8, 8.6)	3.9 (−3.0, 10.8)
Women	2.1 (−3.9, 8.0)	−2.2 (−7.0, 2.6)	1.7 (−3.7, 7.2)
Total	5.5 (0.2, 10.8)^*^	−0.4 (−5.5, 4.7)	5.6 (0, 11.3)^*^
**45–64 yrs**			
Men	10.0 (5.4, 14.5)^*^	10.7 (6.5, 14.8)^*^	12.0 (8.1, 15.9)^*^
Women	4.8 (−0.1, 9.7)	9.9 (5.3, 14.5)^*^	9.2 (5.1, 13.3)^*^
Total	11.8 (7.7, 15.8)^*^	10.5 (7.1, 13.9)^*^	10.7 (7.5, 14.0)^*^
**≥65 yrs**			
Men	−3.6 (−8.9, 1.7)	5.1 (2.8, 7.4)^*^	2.7 (0.5, 4.9)^*^
Women	3.0 (−2.0, 7.9)	7.4 (2.2, 12.6)^*^	6.4 (1.8, 10.9)^*^
Total	−0.8 (−5.5, 3.9)	6.2 (3.4, 9.0)^*^	4.3 (1.7, 7.0)^*^

* *P < 0.05*.

### Association of Macroeconomic Development With Stroke Incidence Over Time

**Figure 2:**
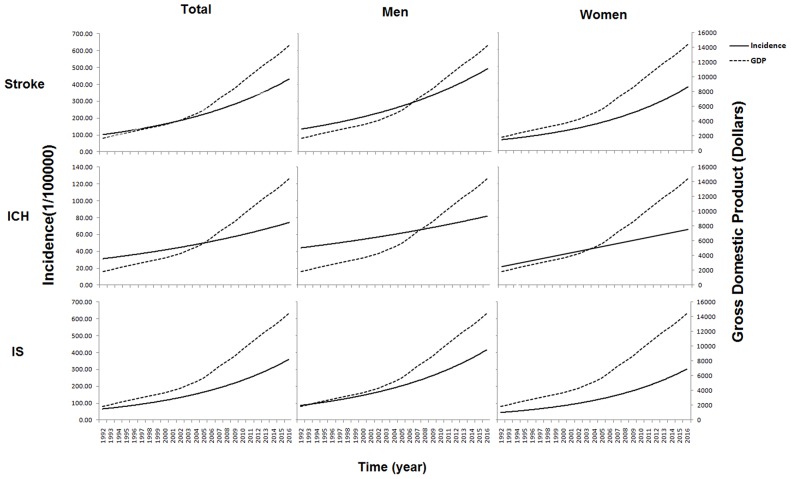
Association of GDP with stroke incidence over time during the 25-year study period. It shows that the first-ever stroke incidence increased by 10.7% per 1,000 dollars increase in PPP-aGDP overall during the 25-year study period, with increases of 9.6% (6.8 to 12.4%) in men and 13.0% (9.0 to 16.9%) in women (all *P* < 0.001). With regard to stroke types, incidence increased by 6.2% (95%CI: 1.6 to 10.8%; *P* = 0.011) for ICH and 12.6% (95%CI: 9.7 to 15.6%; *P* < 0.001) for IS following 1,000 dollars increasing of PPP-aGDP.

The overall first-ever stroke incidence increased by 10.7% per 1,000 USD increase in PPP-aGDP during the 25-year study period, with mean increases of 9.6% in men and 13.0% in women (all *P* < 0.001). Moreover, for every 1,000 USD increase in PPP-aGDP, the overall incidence of ICH increased by 6.2% (*P* = 0.011) and that of IS increased by 12.6% (*P* < 0.001; Figure 2).

Over the same period, a similar trend was found in the association of PCNI with stroke incidence across sex, age group, and types. Overall, the incidence of first-ever stroke increased by 12.0% per 1,000 Yuan increase in the PCNI (10.8% in men and 14.4% in women, *P* < 0.001). Greater increases in incidence were observed for IS than for ICH, as well as for patients ≥65-years old compared with those in younger age categories (Table 4).

**Table 4:** The association of stroke incidence with macroeconomic development by sex, age, and subtypes (β with 95% CI).

**Groups**	**PPP-aGDP**			**PCNI**		
	**β**	**95% CI**	***P***	**β**	**95% CI**	***P***
**INCIDENCES**						
**Gender**						
Men	0.096	0.068 to 0.124	<0.001	0.108	0.069 to 0.147	<0.001
Women	0.130	0.090 to 0.169	<0.001	0.144	0.089 to 0.199	<0.001
Total	0.107	0.078 to 0.136	<0.001	0.120	0.079 to 0.161	<0.001
**Types**						
ICH	0.062	0.016 to 0.108	<0.001	0.062	0.004 to 0.121	0.039
IS	0.126	0.097 to 0.156	0.005	0.143	0.101 to 0.185	<0.001
**Age groups**						
<65 yrs	0.050	0.015 to 0.085	0.007	0.057	0.013 to 0.100	0.013
≥65 yrs	0.065	0.022 to 0.109	0.005	0.073	0.019 to 0.127	0.010
**AGE OF STROKE ONSET**						
**Total**						
Stroke	−0.189	−0.459 to 0.081	0.160	−0.218	−0.545 to 0.108	0.180
ICH	−0.480	−0.889 to −0.072	0.023	−0.506	−1.014 to 0.002	0.051
IS	−0.150	−0.458 to 0.159	0.325	−0.192	−0.563 to 0.179	0.296
**Men**						
Stroke	−0.383	−0.669 to −0.097	0.011	−0.431	−0.783 to −0.079	0.019
ICH	−0.957	−1.508 to −0.406	0.002	−1.080	−1.767 to −0.394	0.003
IS	−0.282	−0.600 to 0.037	0.081	−0.331	−0.717 to 0.054	0.089
**Women**						
Stroke	−0.118	−0.907 to 0.671	0.760	−0.111	−1.063 to 0.840	0.811
ICH	0.343	−0.200 to 0.886	0.204	0.442	−0.205 to 1.090	0.171
IS	0.114	−0.424 to 0.652	0.665	0.075	−0.575 to 0.725	0.813

### Association of GDP With Age at Stroke Onset Over Time

The age at stroke onset varied with increases in PPP-aGDP. There was a 0.48-year decrease in the age of ICH onset (*P* = 0.023) for the overall study population; the most significant decrease was a 0.96-year decrease in the age of ICH onset in men (*P* = 0.002; Table 4). There was a significant association of increased PCNI with a younger age of stroke onset in men experiencing ICHs and first-ever strokes; the same association was not evident in women. The age of stroke onset in men decreased by 0.43 (*P* = 0.016, stroke) and 1.08 (*P* = 0.003, ICH) years for each 1,000 Yuan increase in PCNI (Table 4).

## Discussion

To our knowledge, this is the first report to quantitatively describe the relationship between national macroeconomic development, measured as PPP-aGDP (in USD) and PCNI (in Yuan), and stroke incidence and age of onset trends over time. The preponderant stroke type was IS in this population (77.6%), with a ratio of IS:ICH of 3.5:1. The annual change in stroke incidence was 6.0% from 1992 to 2016 in a rural population in China. The age-standardized incidence of first-ever stroke increased in concert with increases in PPP-aGDP and PCNI for both sexes, all stroke types, and age categories over the 25-year study period. Each 1,000 USD increase in PPP-aGDP generated a 10.7% increase in stroke incidence overall, with rates of 9.6% for men, 13.0% for women, 6.2% for ICH, and 12.6% for IS. Further, patients <65-years old experienced a 5.0% increase, and those ≥65-years old experienced a 6.5% increase. Corresponding increases were also observed for each 1,000 Yuan increase in PCNI. Moreover, these PPP-aGDP and PCNI increases were associated with earlier ICH onset in men; the age of ICH onset decreased by 0.96- and 1.08-years, respectively.

Over the past decades, stroke incidence and mortality have decreased in high-income countries, but increased in low to-middle-income countries (1). Concurrently, IS has been established as the most common type of stroke (26), although a higher incidence of hemorrhagic stroke has been observed in Asian populations, especially in China (27, 28). Consistent with previous studies, we reported that IS was the preponderant stroke type, accounting for 77.6% of all strokes. Following rapid economic development, life conditions have evidently improved in China, especially among rural residents. Concurrently, the prevalence of atherosclerotic risk factors has also increased, which consequently increased the incidence of IS, which is categorized as a large-artery atherosclerosis stroke (29). The increased occurrence of large-artery atherosclerosis stroke may partly explain the higher proportion of IS in this study.

Previous studies demonstrated that Chinese populations had a higher proportion of ICH than that in Caucasian populations (27, 28, 30–32). In addition, ICH accounted for a larger, more variable proportion of strokes in China than in Taiwan (27–51% vs. 17–28%) and in community-based Chinese than Caucasians (pooled proportion 33% vs. 12%) (33). Furthermore, there were striking variations of the ICH proportion, even in China. In addition, the Sino-MONICA-Beijing study reported a large reduction in the proportion of incident strokes attributed to hemorrhagic stroke in 2004 (43% in 1984 vs. 14%) (8). A nationwide stroke survey conducted in 2013 showed that for all strokes, IS constituted 69.6%, ICH 23.8%, subarachnoid hemorrhage 4.4%, and undetermined type 2.1% (10). Similar to these recent studies, we observed a IS/ICH ratio of 3.5:1, with ICH comprising 22.4%. However, the ICH proportion was obviously lower in this population than in other populations (27). Lower socioeconomic status and different types of hemorrhagic stroke (regardless of inclusion of SAH) may have contributed to the lower proportion of ICH observed in the current study.

The stroke burden in China has increased over the past 30-years and remains particularly high in rural areas (10). Previous studies in Hong Kong and Changsha reported significant increases in IS incidence but no change in ICH incidence (9, 34). First-ever stroke incidence has also increased over the last two decades among rural residents (11, 12). The steady increase in stroke incidence associated with the current study population may be partly attributed to the dramatic increases in the prevalence of the stroke risk factors described below.

Low socioeconomic status has been associated with a high stroke risk and first-ever stroke incidence, despite the use of different measurement methodologies (17, 35–37). A systematic review indicated that PPP-aGDP is negatively associated with stroke incidence, 30-day case-fatality rates, and the proportion of patients experiencing ICH; accordingly, populations with low PPP-aGDP levels are at greater risk of stroke and have younger ages of stroke onset than populations with high PPP-aGDP levels (18). Another study demonstrated a higher incidence of first-ever stroke, greater early mortality rates, and a larger proportion of hemorrhagic strokes among patients from low- to middle-income countries than among those from higher-income countries (38). Lower national income levels have also been associated with higher relative mortality rates and greater stroke burdens (39).

Poverty has long been known to affect rates of hypertension, diabetes mellitus, obesity, heart attacks, and strokes (40, 41). A recent cohort study of relatively young adults demonstrated that income volatility and decreases in income during the 15-year period of formative earning years were independently associated with a nearly 2-fold increase in the risk of cardiovascular disease and all-cause mortality (42). These previous studies transversely assessed stroke burden by comparing different economic levels between countries or populations. However, in the present study, we investigated longitudinal associations between national macroeconomic levels and trends in first-ever stroke incidence. We found a positive association of PPP-aGDP and PCNI with first-ever stroke incidence in a rural population in China. Each 1,000 USD increase in PPP-aGDP and each 1,000 Yuan increase in PCNI correlated with a 10.7 and 12.0% increase, respectively, in age-standardized incidence of first-ever stroke, irrespective of sex, age, or stroke type. Additionally, these same increases in PPP-aGDP and PCNI were also associated with a younger age (0.96- and 1.08-year decrease, respectively) of ICH onset in men.

Over the past 40-years, rapid economic development in China has generated an extensive shift in the level of agricultural mechanization, resulting in a significant decrease in physical labor. Simultaneously, more foods have become available in conjunction with this economic development, especially in rural areas. Furthermore, lifestyle and diet changes have significantly altered the traditional diets of rural residents, who now favor diets low in fruits, high in sodium, and low in whole grains, nuts, and seeds (40). Documents from China's Ministry of Health show that the energy ratio obtained from cereal decreased by 15.3% but the energy ratios derived from animal protein and fat increased by 87.1 and 48.9%, respectively, among rural residents between 1992 and 2002 (43). Our previous studies also revealed that the prevalence of hypertension and obesity significantly increased in this rural population over the past two decades (20, 24). These lifestyle and dietary changes may partly explain the upward trend in stroke incidence observed in this study.

Between 1991 and 2011, increases in the prevalence of hypertension (52%), obesity (80%), diabetes (27%), and alcohol consumption (47%) were observed in young and middle-aged individuals (12). Moreover, the average systolic and diastolic blood pressure levels increased by 11.3 and 5.7 mmHg, respectively, in men aged 45–64-years. Such steep increases in the prevalence of these stroke risk factors, in addition to increases in fasting glucose, total cholesterol, and triglyceride levels, are probably the main reasons for the observed decreases in the age of stroke onset (12). Moreover, disparities between the rapid economic development and the less rapid increases in health-care investments may have had consequences on the normal management of conventional stroke risk factors (40).

Although this report demonstrates the magnitude of the relationship between stroke incidence and national economic development, the study has several limitations. First, the study population was from a single township in northern China, which is not representative of the overall Chinese population. However, the prospective study design and long study period may have reduced the negative impact of the study's narrow focus. Additionally, these findings may sound a warning with regards to other populations undergoing economic development, although further studies are needed to determine whether this is valid for other populations. Second, as we were unable to assess individual incomes, we assessed the effects of the national net income for rural residents in China on the incidence of stroke. This may under- or overestimate the relationship between economic development and stroke incidence. Third, the extensive application of neuroimaging during the last decade of the study period may have contributed to the apparent increase in stroke incidence over time. However, all stroke patients in this study experienced symptomatic stroke events (silent strokes and transient ischemic attacks were excluded), which likely reduced any bias due to the increased diagnosis of stroke events. Moreover, the application of national medical insurance might have increased the proportion of neuroimaging-based diagnoses of stroke events as well as the proportion of hospital admissions. However, such medical insurance would not have increased the numbers of stroke events; all stroke events in this study were symptomatic and silent strokes were excluded. Furthermore, the frequency of ischemic stroke may have increased because of increased use of brain magnetic resonance imaging. Finally, the different standardized populations used to assess the standard incidence may have affected the estimate's precision when comparing the incidences observed in this study with those of other studies. However, we discussed stroke incidence trends following variations in national economic development, potentially reducing the impact of the different reference populations. Nonetheless, a nationwide study is needed to determine the overall impact of national economic development on stroke burden.

## Conclusions

We quantitatively demonstrated the association between first-ever stroke incidence and economic development within a rural population in China over a 25-year period. A positive association was observed between age-standardized incidence of first-ever stroke and both PPP-aGDP and PCNI, irrespective of sex, age, or stroke type. The age of stroke onset was also demonstrated to decrease concurrently with increases in PPP-aGDP and PCNI. Decreased physical labor, excess food supply, and westernized lifestyles arising from the rapid economic development in the region resulted in significant increases in the prevalence of stroke risk factors (hypertension, obesity, diabetes, and alcohol consumption) among the residents, especially among young men. These findings suggest the critical need for enhanced health-care investment during periods of remarkable economic development occurring. Such investments are crucial to containing the increasing prevalence of stroke risk factors. Moreover, our findings provide useful reference values for other developing countries and emphasize the need for establishing an effective management strategy for stroke risk factors during the initial stages of economic development.

## Data Availability Statement

The datasets generated for this study are available on request to the corresponding author.

## Ethics Statement

The studies involving human participants were reviewed and approved by The ethics committee of Tianjin Medical University General Hospital. The patients/participants provided their written informed consent to participate in this study.

## Author Contributions

XN, JW, and YW were involved in conception and design and data interpretation for this article. JW was involved in data analysis for this article. PZ and JL were involved in manuscript drafting. PZ, JL, YH, QL, YG, JT, and JW were involved in data collection, case diagnosis, and confirmation for this article. XN, JW, and YW were involved in critical review for this article.

## Conflict of Interest

The authors declare that the research was conducted in the absence of any commercial or financial relationships that could be construed as a potential conflict of interest.
