# Supplementary material for: Macroeconomic Development and Dramatic Increase in Stroke Burden in Rural China: A 25-Year Population-Based Study
Source: Front Neurol. 2020 May 13;11:385. doi: 10.3389/fneur.2020.00385 (PMC7237581; doi:10.3389/fneur.2020.00385)
Supplement: Supplementary file 1 [file Table_1.DOC]

Supplemental Table 1. Distribution of population by age and sex in the Tianjin Brain Study during 1992 to 2016 (%).

| Age group | P1 | P2 | P3 | P4 | P5 |
| --- | --- | --- | --- | --- | --- |
| Men: |  |  |  |  |  |
| <35 years | 46.6 | 47.1 | 47.7 | 46.7 | 46.0 |
| 35~44 years | 13.4 | 13.2 | 12.7 | 12.7 | 12.5 |
| 45~54 years | 15.5 | 15.3 | 15.0 | 15.5 | 15.0 |
| 55~64 years | 14.7 | 14.4 | 14.0 | 14.5 | 16.0 |
| 65~74 years | 6.5 | 6.6 | 6.8 | 6.7 | 6.6 |
| ≥ 75 years | 3.3 | 3.5 | 3.8 | 3.8 | 3.9 |
| Women: |  |  |  |  |  |
| <35 years | 39.8 | 39.8 | 39.0 | 39.0 | 38.5 |
| 35~44 years | 14.6 | 14.7 | 14.6 | 14.6 | 13.9 |
| 45~54 years | 17.5 | 17.6 | 17.8 | 18.0 | 17.7 |
| 55~64 years | 17.6 | 16.5 | 16.9 | 16.8 | 18.1 |
| 65~74 years | 6.8 | 6.8 | 6.8 | 6.6 | 6.8 |
| ≥ 75 years | 3.8 | 4.6 | 4.9 | 5.1 | 5.1 |
| Total: |  |  |  |  |  |
| <35 years | 43.3 | 43.6 | 43.5 | 43.0 | 42.5 |
| 35~44 years | 14.0 | 13.9 | 13.6 | 13.6 | 13.2 |
| 45~54 years | 16.5 | 16.5 | 16.4 | 16.7 | 16.2 |
| 55~64 years | 16.1 | 15.4 | 15.4 | 15.6 | 17.0 |
| 65~74 years | 6.6 | 6.7 | 6.8 | 6.6 | 6.7 |
| ≥ 75 years | 3.5 | 4.0 | 4.4 | 4.4 | 4.5 |

P1: 1992-1996, P2: 1997-2001, P3: 2002-2006, P4: 2007-2011, P5: 2012-2016.
