# Supplementary material for: Macroeconomic Development and Dramatic Increase in Stroke Burden in Rural China: A 25-Year Population-Based Study
Source: Front Neurol. 2020 May 13;11:385. doi: 10.3389/fneur.2020.00385 (PMC7237581; doi:10.3389/fneur.2020.00385)
Supplement: Supplementary file 2 [file Table_2.DOCX]

Supplemental Table 2. The conversion rate of exchanged USD for RMB and PCNI during 1992 to 2016

| Year | Conversion rate | PCNI (RMB) | PCNI (USD) |
| --- | --- | --- | --- |
| 1992 | 5.516 | 784.0 | 142.1 |
| 1993 | 5.762 | 921.6 | 159.9 |
| 1994 | 8.619 | 1221.0 | 141.7 |
| 1995 | 8.351 | 1577.7 | 188.9 |
| 1996 | 8.314 | 1926.1 | 231.7 |
| 1997 | 8.290 | 2090.1 | 252.1 |
| 1998 | 8.279 | 2162.0 | 261.1 |
| 1999 | 8.278 | 2210.3 | 267.0 |
| 2000 | 8.279 | 2253.4 | 272.2 |
| 2001 | 8.277 | 2366.4 | 285.9 |
| 2002 | 8.277 | 2475.6 | 299.1 |
| 2003 | 8.277 | 2622.2 | 316.8 |
| 2004 | 8.0922 | 2936.4 | 362.9 |
| 2005 | 8.1917 | 3254.9 | 397.3 |
| 2006 | 7.9735 | 3587.0 | 451.9 |
| 2007 | 7.5215 | 4141.4 | 550.6 |
| 2008 | 6.9385 | 4760.6 | 686.1 |
| 2009 | 6.83 | 5153.2 | 754.5 |
| 2010 | 6.615 | 5919.0 | 894.8 |
| 2011 | 6.3851 | 6977.3 | 1092.7 |
| 2012 | 6.298 | 7916.6 | 1257.0 |
| 2013 | 6.193 | 8895.9 | 1436.4 |
| 2014 | 6.1704 | 9892.0 | 1603.1 |
| 2015 | 6.2284 | 10772.0 | 1729.5 |
| 2016 | 6.6423 | 12363.4 | 1861.3 |
